# 2-{2-[3-(1*H*-Benzimidazol-2-yl)quinolin-2-yl­oxy]eth­oxy}ethanol

**DOI:** 10.1107/S1600536809002797

**Published:** 2009-01-28

**Authors:** Frank Rominger, Mahalingam Malathi, Palathurai Subramaniam Mohan, Tanuja Ramamurthi Dondeti, A. Stephen K. Hashmi

**Affiliations:** a Organisch-Chemisches Institut, Universität Heidelberg, Im Neuenheimer Feld 270, 69120 Heidelberg, Germany; b Department of Chemistry, Bharathiar University, Coimbatore 641 046, India

## Abstract

In the title compound, C_20_H_19_N_3_O_3_, the inter­planar angle between the benzimidazole unit and the quinoline unit is 25.1 (2)°. Two different hydrogen bonds involving the hydr­oxy group and the imidazole unit are present. An intra­molecular N—H⋯O hydrogen bond links the hydr­oxy group of the side chain with the imidazole unit, forming a 12-membered ring, and an inter­molecular O—H⋯N hydrogen bond links the mol­ecules, forming chains in the crystallographic *b* direction.

## Related literature

A closely related structure is reported in the previous paper, see: Rominger *et al.* (2009 ▶). An analogous pyridine compound is essentially flat (Kim *et al.*, 2005 ▶).
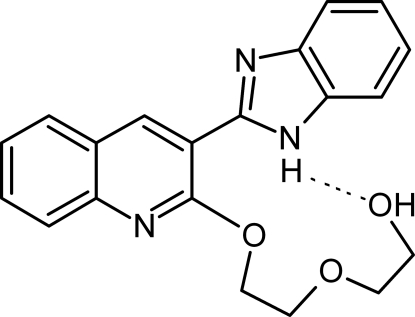


## Experimental

### 

#### Crystal data

C_20_H_19_N_3_O_3_
*M* *_r_* = 349.38
Monoclinic, 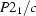

*a* = 11.9478 (2) Å
*b* = 13.1338 (1) Å
*c* = 12.4031 (2) Å
β = 118.744 (1)°
*V* = 1706.47 (4) Å^3^
*Z* = 4
Mo *K*α radiation
μ = 0.09 mm^−1^
*T* = 200 (2) K
0.43 × 0.28 × 0.18 mm

#### Data collection

Bruker SMART CCD diffractometer
Absorption correction: multi-scan (**SADABS**; Sheldrick, 2008*b* ▶) *T*_min_ = 0.960, *T*_max_ = 0.983
16400 measured reflections
3920 independent reflections
2890 reflections with *I* > 2σ(*I*)
*R*_int_ = 0.051

#### Refinement

*R*[*F* ^2^ > 2σ(*F* ^2^)] = 0.044
*wR*(*F* ^2^) = 0.112
*S* = 1.02
3920 reflections
243 parameters
H atoms treated by a mixture of independent and constrained refinement
Δρ_max_ = 0.19 e Å^−3^
Δρ_min_ = −0.18 e Å^−3^

### 

Data collection: *SMART* (Siemens, 1996 ▶); cell refinement: *SAINT* (Siemens, 1996 ▶); data reduction: *SAINT*; program(s) used to solve structure: *SHELXTL* (Sheldrick, 2008*a* ▶); program(s) used to refine structure: *SHELXTL*; molecular graphics: *SHELXTL*; software used to prepare material for publication: *SHELXTL*.

## Supplementary Material

Crystal structure: contains datablocks I, global. DOI: 10.1107/S1600536809002797/fj2184sup1.cif

Structure factors: contains datablocks I. DOI: 10.1107/S1600536809002797/fj2184Isup2.hkl

Additional supplementary materials:  crystallographic information; 3D view; checkCIF report

**Fig. 3.**
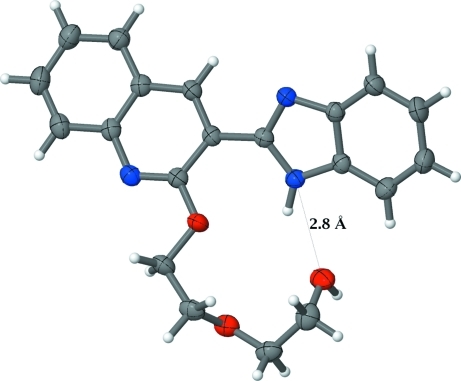
Enhanced figure of the title compound with displacement ellipsoids plotted at 50% probability level.

Enhanced figure: interactive version of Fig. 3

## Figures and Tables

**Table 1:** Hydrogen-bond geometry (Å, °)

*D*—H⋯*A*	*D*—H	H⋯*A*	*D*⋯*A*	*D*—H⋯*A*
N14—H14⋯O37	0.92 (2)	2.04 (2)	2.797 (2)	138.3 (19)
O37—H37⋯N13^i^	0.94 (3)	1.83 (3)	2.765 (2)	175 (2)

Symmetry code: (i) 
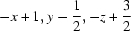
.

**Fig. 1.**
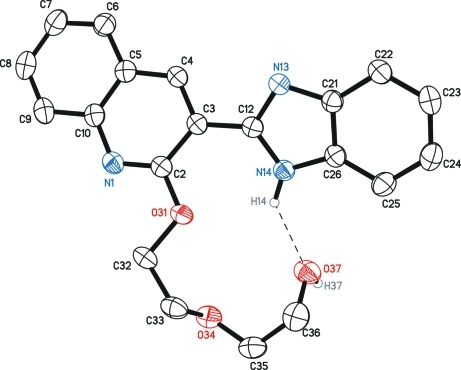
Thermal ellipsoid representation of the title compound with the intramolecular hydrogen bond indicated as dashed line. Displacement ellipsoids were plotted at 50% probability level.

**Fig. 2.**
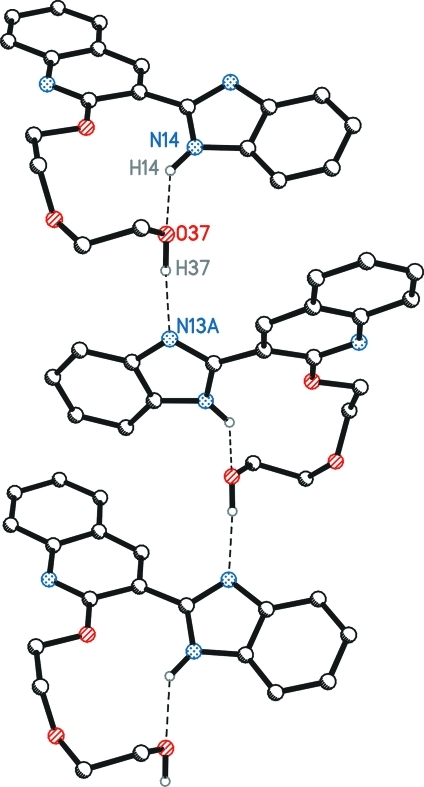
Ball and stick representation of the hydrogen bond connected chain along the b direction. Hydrogen atoms not involved in hydrogen bonds (dashed lines) have been omitted.

## supplementary crystallographic information

### Refinement

For all hydrogen atoms bond to a carbon atom the positions were calculated according to geometrical criteria. During the refinement the hydrogen atoms were allowed to shift with the preceding carbon atoms. The isotropic displacement parameters were set as 1.2 times the equivalent isotropic displacement parameters of the preceding carbon atoms. The positions of two hydrogen atoms of the heteroatoms were refined isotropically.

### Crystal data

**Table:**

C_20_H_19_N_3_O_3_	*F*(000) = 736
*M**_r_* = 349.38	*D*_x_ = 1.360 Mg m^−^^3^
Monoclinic, *P*2_1_/*c*	Mo *K*α radiation, λ = 0.71073 Å
Hall symbol: -P 2ybc	Cell parameters from 7839 reflections
*a* = 11.9478 (2) Å	θ = 1.9–27.5°
*b* = 13.1338 (1) Å	µ = 0.09 mm^−^^1^
*c* = 12.4031 (2) Å	*T* = 200 K
β = 118.744 (1)°	Polyhedron, colourless
*V* = 1706.47 (4) Å^3^	0.43 × 0.28 × 0.18 mm
*Z* = 4	

### Data collection

**Table:**

Bruker SMART CCD diffractometer	3920 independent reflections
Radiation source: fine-focus sealed tube	2890 reflections with *I* > 2σ(*I*)
graphite	*R*_int_ = 0.051
ω scans	θ_max_ = 27.5°, θ_min_ = 1.9°
Absorption correction: multi-scan (*SADABS*; Sheldrick, 2008b)	*h* = −15→15
*T*_min_ = 0.960, *T*_max_ = 0.983	*k* = −16→17
16400 measured reflections	*l* = −16→16

### Refinement

**Table:**

Refinement on *F*^2^	Primary atom site location: structure-invariant direct methods
Least-squares matrix: full	Secondary atom site location: difference Fourier map
*R*[*F*^2^ > 2σ(*F*^2^)] = 0.044	Hydrogen site location: inferred from neighbouring sites
*wR*(*F*^2^) = 0.112	H atoms treated by a mixture of independent and constrained refinement
*S* = 1.02	*w* = 1/[σ^2^(*F*_o_^2^) + (0.0424*P*)^2^ + 0.5312*P*] where *P* = (*F*_o_^2^ + 2*F*_c_^2^)/3
3920 reflections	(Δ/σ)_max_ = 0.001
243 parameters	Δρ_max_ = 0.19 e Å^−^^3^
0 restraints	Δρ_min_ = −0.18 e Å^−^^3^

### Special details

**Table:**

Geometry. All e.s.d.'s (except the e.s.d. in the dihedral angle between two l.s. planes) are estimated using the full covariance matrix. The cell e.s.d.'s are taken into account individually in the estimation of e.s.d.'s in distances, angles and torsion angles; correlations between e.s.d.'s in cell parameters are only used when they are defined by crystal symmetry. An approximate (isotropic) treatment of cell e.s.d.'s is used for estimating e.s.d.'s involving l.s. planes.
Refinement. Refinement of *F*^2^ against ALL reflections. The weighted *R*-factor *wR* and goodness of fit *S* are based on *F*^2^, conventional *R*-factors *R* are based on *F*, with *F* set to zero for negative *F*^2^. The threshold expression of *F*^2^ > σ(*F*^2^) is used only for calculating *R*-factors(gt) *etc*. and is not relevant to the choice of reflections for refinement. *R*-factors based on *F*^2^ are statistically about twice as large as those based on *F*, and *R*- factors based on ALL data will be even larger.

### Fractional atomic coordinates and isotropic or equivalent isotropic displacement parameters (Å^2^)

**Table:**

	*x*	*y*	*z*	*U*_iso_*/*U*_eq_	
N1	0.88443 (12)	0.06208 (10)	0.82383 (11)	0.0329 (3)	
C2	0.76960 (14)	0.02903 (11)	0.79014 (13)	0.0292 (3)	
C3	0.70017 (13)	0.04708 (11)	0.85560 (13)	0.0280 (3)	
C4	0.76217 (14)	0.10173 (11)	0.96231 (13)	0.0296 (3)	
H4	0.7203	0.1149	1.0094	0.036*	
C5	0.88674 (14)	0.13885 (11)	1.00364 (13)	0.0313 (3)	
C6	0.95367 (16)	0.19686 (13)	1.11232 (15)	0.0390 (4)	
H6	0.9141	0.2129	1.1608	0.047*	
C7	1.07435 (17)	0.22987 (14)	1.14778 (16)	0.0453 (4)	
H7	1.1192	0.2679	1.2216	0.054*	
C8	1.13282 (17)	0.20803 (14)	1.07591 (17)	0.0452 (4)	
H8	1.2171	0.2315	1.1015	0.054*	
C9	1.06998 (16)	0.15334 (13)	0.96934 (16)	0.0401 (4)	
H9	1.1105	0.1396	0.9212	0.048*	
C10	0.94551 (14)	0.11733 (11)	0.93079 (14)	0.0315 (3)	
C12	0.56784 (13)	0.01642 (11)	0.81376 (13)	0.0276 (3)	
N13	0.49537 (12)	0.06596 (9)	0.85027 (11)	0.0306 (3)	
N14	0.50501 (12)	−0.06326 (9)	0.73809 (11)	0.0296 (3)	
H14	0.5367 (18)	−0.1089 (15)	0.7031 (17)	0.053 (5)*	
C21	0.37911 (14)	0.01587 (11)	0.79551 (13)	0.0300 (3)	
C22	0.26652 (15)	0.03860 (13)	0.79847 (15)	0.0376 (4)	
H22	0.2627	0.0936	0.8463	0.045*	
C23	0.16163 (16)	−0.02100 (14)	0.73012 (16)	0.0427 (4)	
H23	0.0837	−0.0065	0.7302	0.051*	
C24	0.16696 (16)	−0.10276 (14)	0.66025 (17)	0.0442 (4)	
H24	0.0926	−0.1427	0.6143	0.053*	
C25	0.27764 (16)	−0.12678 (13)	0.65644 (15)	0.0395 (4)	
H25	0.2813	−0.1825	0.6093	0.047*	
C26	0.38342 (14)	−0.06551 (11)	0.72491 (13)	0.0296 (3)	
O31	0.70752 (10)	−0.02559 (8)	0.68516 (9)	0.0339 (3)	
C32	0.77582 (16)	−0.04159 (13)	0.61680 (14)	0.0381 (4)	
H32A	0.8550	−0.0808	0.6669	0.046*	
H32B	0.7993	0.0246	0.5951	0.046*	
C33	0.68995 (17)	−0.09925 (12)	0.50313 (14)	0.0396 (4)	
H33A	0.6050	−0.0662	0.4615	0.048*	
H33B	0.7258	−0.0988	0.4459	0.048*	
O34	0.67706 (11)	−0.20130 (8)	0.53374 (10)	0.0383 (3)	
C35	0.56108 (17)	−0.24765 (13)	0.44759 (15)	0.0427 (4)	
H35A	0.5703	−0.3225	0.4563	0.051*	
H35B	0.5436	−0.2298	0.3634	0.051*	
C36	0.44979 (17)	−0.21515 (14)	0.46430 (17)	0.0470 (4)	
H36A	0.4319	−0.1422	0.4429	0.056*	
H36B	0.3732	−0.2544	0.4074	0.056*	
O37	0.47303 (13)	−0.23023 (9)	0.58596 (12)	0.0464 (3)	
H37	0.486 (2)	−0.300 (2)	0.604 (2)	0.077 (7)*	

### Atomic displacement parameters (Å^2^)

**Table:**

	*U*^11^	*U*^22^	*U*^33^	*U*^12^	*U*^13^	*U*^23^
N1	0.0349 (7)	0.0330 (7)	0.0346 (7)	0.0019 (5)	0.0197 (6)	0.0033 (5)
C2	0.0315 (8)	0.0271 (7)	0.0299 (7)	0.0032 (6)	0.0157 (6)	0.0034 (6)
C3	0.0296 (8)	0.0261 (7)	0.0301 (7)	0.0030 (6)	0.0158 (6)	0.0042 (6)
C4	0.0307 (8)	0.0295 (8)	0.0315 (7)	0.0029 (6)	0.0173 (6)	0.0029 (6)
C5	0.0325 (8)	0.0282 (8)	0.0320 (8)	0.0028 (6)	0.0145 (6)	0.0048 (6)
C6	0.0402 (9)	0.0388 (9)	0.0379 (9)	−0.0041 (7)	0.0187 (7)	−0.0019 (7)
C7	0.0440 (10)	0.0421 (10)	0.0424 (9)	−0.0114 (8)	0.0148 (8)	−0.0034 (8)
C8	0.0362 (9)	0.0430 (10)	0.0527 (11)	−0.0087 (8)	0.0183 (8)	0.0033 (8)
C9	0.0367 (9)	0.0393 (9)	0.0490 (10)	−0.0004 (7)	0.0243 (8)	0.0067 (8)
C10	0.0312 (8)	0.0280 (8)	0.0354 (8)	0.0025 (6)	0.0160 (7)	0.0058 (6)
C12	0.0308 (8)	0.0264 (7)	0.0261 (7)	0.0022 (6)	0.0142 (6)	0.0038 (6)
N13	0.0304 (7)	0.0323 (7)	0.0318 (6)	−0.0010 (5)	0.0170 (5)	−0.0026 (5)
N14	0.0334 (7)	0.0249 (6)	0.0337 (7)	0.0006 (5)	0.0187 (6)	−0.0003 (5)
C21	0.0309 (8)	0.0309 (8)	0.0289 (7)	−0.0010 (6)	0.0150 (6)	0.0021 (6)
C22	0.0351 (9)	0.0420 (9)	0.0395 (8)	0.0011 (7)	0.0210 (7)	−0.0023 (7)
C23	0.0332 (9)	0.0493 (10)	0.0499 (10)	−0.0028 (8)	0.0235 (8)	0.0006 (8)
C24	0.0368 (9)	0.0440 (10)	0.0502 (10)	−0.0104 (8)	0.0196 (8)	−0.0047 (8)
C25	0.0434 (9)	0.0322 (8)	0.0438 (9)	−0.0060 (7)	0.0217 (8)	−0.0053 (7)
C26	0.0322 (8)	0.0275 (7)	0.0310 (7)	0.0004 (6)	0.0167 (6)	0.0041 (6)
O31	0.0359 (6)	0.0396 (6)	0.0318 (5)	0.0000 (5)	0.0207 (5)	−0.0043 (5)
C32	0.0433 (9)	0.0443 (9)	0.0367 (8)	0.0026 (7)	0.0272 (7)	0.0004 (7)
C33	0.0553 (10)	0.0382 (9)	0.0331 (8)	0.0045 (8)	0.0274 (8)	0.0049 (7)
O34	0.0460 (7)	0.0355 (6)	0.0343 (6)	0.0061 (5)	0.0199 (5)	0.0039 (5)
C35	0.0560 (11)	0.0388 (9)	0.0333 (8)	0.0006 (8)	0.0214 (8)	−0.0013 (7)
C36	0.0478 (10)	0.0397 (10)	0.0505 (11)	0.0007 (8)	0.0213 (9)	0.0065 (8)
O37	0.0656 (8)	0.0295 (6)	0.0596 (8)	−0.0021 (6)	0.0425 (7)	−0.0045 (6)

### Geometric parameters (Å, °)

**Table:**

N1—C2	1.3015 (19)	C21—C26	1.398 (2)
N1—C10	1.374 (2)	C22—C23	1.371 (2)
C2—O31	1.3532 (17)	C22—H22	0.9500
C2—C3	1.432 (2)	C23—C24	1.400 (3)
C3—C4	1.368 (2)	C23—H23	0.9500
C3—C12	1.464 (2)	C24—C25	1.382 (2)
C4—C5	1.408 (2)	C24—H24	0.9500
C4—H4	0.9500	C25—C26	1.391 (2)
C5—C10	1.414 (2)	C25—H25	0.9500
C5—C6	1.414 (2)	O31—C32	1.4472 (18)
C6—C7	1.360 (2)	C32—C33	1.491 (2)
C6—H6	0.9500	C32—H32A	0.9900
C7—C8	1.401 (3)	C32—H32B	0.9900
C7—H7	0.9500	C33—O34	1.4215 (19)
C8—C9	1.368 (2)	C33—H33A	0.9900
C8—H8	0.9500	C33—H33B	0.9900
C9—C10	1.407 (2)	O34—C35	1.417 (2)
C9—H9	0.9500	C35—C36	1.503 (3)
C12—N13	1.3248 (18)	C35—H35A	0.9900
C12—N14	1.3646 (19)	C35—H35B	0.9900
N13—C21	1.3843 (19)	C36—O37	1.411 (2)
N14—C26	1.3818 (19)	C36—H36A	0.9900
N14—H14	0.92 (2)	C36—H36B	0.9900
C21—C22	1.396 (2)	O37—H37	0.94 (3)
			
C2—N1—C10	118.33 (13)	C21—C22—H22	121.1
N1—C2—O31	119.41 (13)	C22—C23—C24	121.35 (16)
N1—C2—C3	124.84 (14)	C22—C23—H23	119.3
O31—C2—C3	115.75 (13)	C24—C23—H23	119.3
C4—C3—C2	116.32 (13)	C25—C24—C23	121.67 (16)
C4—C3—C12	118.51 (13)	C25—C24—H24	119.2
C2—C3—C12	125.09 (13)	C23—C24—H24	119.2
C3—C4—C5	121.31 (14)	C24—C25—C26	116.78 (15)
C3—C4—H4	119.3	C24—C25—H25	121.6
C5—C4—H4	119.3	C26—C25—H25	121.6
C4—C5—C10	117.46 (14)	N14—C26—C25	132.82 (14)
C4—C5—C6	123.21 (14)	N14—C26—C21	105.24 (13)
C10—C5—C6	119.33 (14)	C25—C26—C21	121.90 (14)
C7—C6—C5	120.36 (16)	C2—O31—C32	115.99 (12)
C7—C6—H6	119.8	O31—C32—C33	107.60 (13)
C5—C6—H6	119.8	O31—C32—H32A	110.2
C6—C7—C8	120.28 (16)	C33—C32—H32A	110.2
C6—C7—H7	119.9	O31—C32—H32B	110.2
C8—C7—H7	119.9	C33—C32—H32B	110.2
C9—C8—C7	120.81 (16)	H32A—C32—H32B	108.5
C9—C8—H8	119.6	O34—C33—C32	109.88 (13)
C7—C8—H8	119.6	O34—C33—H33A	109.7
C8—C9—C10	120.26 (16)	C32—C33—H33A	109.7
C8—C9—H9	119.9	O34—C33—H33B	109.7
C10—C9—H9	119.9	C32—C33—H33B	109.7
N1—C10—C9	119.30 (14)	H33A—C33—H33B	108.2
N1—C10—C5	121.74 (13)	C35—O34—C33	113.32 (12)
C9—C10—C5	118.95 (15)	O34—C35—C36	112.53 (14)
N13—C12—N14	112.21 (13)	O34—C35—H35A	109.1
N13—C12—C3	121.05 (13)	C36—C35—H35A	109.1
N14—C12—C3	126.73 (13)	O34—C35—H35B	109.1
C12—N13—C21	105.40 (12)	C36—C35—H35B	109.1
C12—N14—C26	107.28 (12)	H35A—C35—H35B	107.8
C12—N14—H14	127.4 (12)	O37—C36—C35	112.06 (15)
C26—N14—H14	125.3 (12)	O37—C36—H36A	109.2
N13—C21—C22	129.64 (14)	C35—C36—H36A	109.2
N13—C21—C26	109.88 (13)	O37—C36—H36B	109.2
C22—C21—C26	120.41 (14)	C35—C36—H36B	109.2
C23—C22—C21	117.88 (15)	H36A—C36—H36B	107.9
C23—C22—H22	121.1	C36—O37—H37	108.8 (14)
			
C10—N1—C2—O31	−179.98 (13)	N14—C12—N13—C21	0.02 (16)
C10—N1—C2—C3	0.9 (2)	C3—C12—N13—C21	−178.94 (12)
N1—C2—C3—C4	−1.2 (2)	N13—C12—N14—C26	−0.11 (16)
O31—C2—C3—C4	179.63 (13)	C3—C12—N14—C26	178.78 (13)
N1—C2—C3—C12	175.39 (14)	C12—N13—C21—C22	−176.95 (16)
O31—C2—C3—C12	−3.7 (2)	C12—N13—C21—C26	0.07 (16)
C2—C3—C4—C5	0.8 (2)	N13—C21—C22—C23	176.58 (15)
C12—C3—C4—C5	−176.08 (13)	C26—C21—C22—C23	−0.2 (2)
C3—C4—C5—C10	−0.1 (2)	C21—C22—C23—C24	0.6 (3)
C3—C4—C5—C6	179.06 (14)	C22—C23—C24—C25	−0.3 (3)
C4—C5—C6—C7	179.50 (15)	C23—C24—C25—C26	−0.3 (3)
C10—C5—C6—C7	−1.3 (2)	C12—N14—C26—C25	177.47 (16)
C5—C6—C7—C8	1.0 (3)	C12—N14—C26—C21	0.15 (15)
C6—C7—C8—C9	0.0 (3)	C24—C25—C26—N14	−176.25 (16)
C7—C8—C9—C10	−0.6 (3)	C24—C25—C26—C21	0.7 (2)
C2—N1—C10—C9	179.66 (14)	N13—C21—C26—N14	−0.14 (16)
C2—N1—C10—C5	−0.2 (2)	C22—C21—C26—N14	177.20 (13)
C8—C9—C10—N1	−179.64 (15)	N13—C21—C26—C25	−177.82 (14)
C8—C9—C10—C5	0.2 (2)	C22—C21—C26—C25	−0.5 (2)
C4—C5—C10—N1	−0.2 (2)	N1—C2—O31—C32	−0.87 (19)
C6—C5—C10—N1	−179.44 (14)	C3—C2—O31—C32	178.30 (13)
C4—C5—C10—C9	179.96 (14)	C2—O31—C32—C33	−177.84 (12)
C6—C5—C10—C9	0.7 (2)	O31—C32—C33—O34	−71.36 (16)
C4—C3—C12—N13	22.5 (2)	C32—C33—O34—C35	153.87 (13)
C2—C3—C12—N13	−154.07 (14)	C33—O34—C35—C36	−78.72 (17)
C4—C3—C12—N14	−156.31 (14)	O34—C35—C36—O37	−53.58 (19)
C2—C3—C12—N14	27.1 (2)		

### Hydrogen-bond geometry (Å, °)

**Table:**

*D*—H···*A*	*D*—H	H···*A*	*D*···*A*	*D*—H···*A*
N14—H14···O37	0.92 (2)	2.04 (2)	2.797 (2)	138.3 (19)
O37—H37···N13^i^	0.94 (3)	1.83 (3)	2.765 (2)	175 (2)

Symmetry codes:  (i) −*x*+1, *y*−1/2, −*z*+3/2.
